# Honey Bee Health in Maine Wild Blueberry Production

**DOI:** 10.3390/insects12060523

**Published:** 2021-06-05

**Authors:** Francis A. Drummond, Jennifer Lund, Brian Eitzer

**Affiliations:** 1 School of Biology and Ecology, University of Maine, Orono, ME 04469, USA; 2 Department of Agriculture, Conservation and Forestry, Augusta, ME 04330, USA; jennifer.lund@maine.gov; 3 Department of Analytical Chemistry, The Connecticut Agricultural Experiment Station, New Haven, CT 06511, USA; brian.eitzer@ct.gov

**Keywords:** Varroa mite, pesticide residues, pathogens, *Apis mellifera*, migratory hives, Sinai virus, *Lotmaria passim*, *Apocephalus borealis*

## Abstract

**Simple Summary:**

Wild blueberry is an important native North American crop that requires insect pollination. Migratory western honey bee colonies constitute the majority of commercial bees brought into Maine for pollination of wild blueberry. Currently, many stressors impact the western honey bee in the US. We designed a two-year monitoring study (2014 and 2015) to assess the potential health of honey bee colonies hired for pollination services in wild blueberry fields. We monitored the colony health of nine hive locations (three hives/location) in 2014 and nine locations (five hives/location) in 2015 during bloom (May–June). Queen health status, colony strength, rate of population increase, and pesticide residues on pollen, wax, and honey bee workers were measured. In addition, each hive was sampled to assess levels of mite parasites, viruses, and Microsporidian and Trypanosome pathogens. Different patterns in colony health were observed over the two years. Factors predicting colony growth rate over both years were Varroa mite infestation and risk due to pollen pesticide residues during bloom. In addition, recently discovered parasites and pathogens were already observed in most of the colonies suggesting that parasites and diseases spread rapidly and become established quickly in commercial honey bee colonies.

**Abstract:**

A two-year study was conducted in Maine wild blueberry fields (*Vaccinium angustifolium* Aiton) on the health of migratory honey bee colonies in 2014 and 2015. In each year, three or five colonies were monitored at each of nine wild blueberry field locations during bloom (mid-May until mid-June). Colony health was measured by assessing colony strength during wild blueberry bloom. Potential factors that might affect colony health were queen failure or supersedure; pesticide residues on trapped pollen, wax comb, and bee bread; and parasites and pathogens. We found that Varroa mite and pesticide residues on trapped pollen were significant predictors of colony health measured as the rate of change in the amount of sealed brood during bloom. These two factors explained 71% of the variance in colony health over the two years. Pesticide exposure was different in each year as were pathogen prevalence and incidence. We detected high prevalence and abundance of two recently discovered pathogens and one recently discovered parasite, the trypanosome *Lotmaria passim* Schwartz, the Sinai virus, and the phorid fly, *Apocephalus borealis* Brues.

## 1. Introduction

Wild blueberries are a northeastern native North American crop that is primarily grown in Maine USA, the Canadian Maritimes, and Quebec, along with limited cropland areas in New Hampshire, Massachusetts, and Michigan USA [1]. The crop is not planted and consists of several wild sympatric *Vaccinium* species along with a hybrid. When a forest is clear-cut, these native understory species flower and produce fruit [1]. In Maine, the most abundant species is *Vaccinium angustifolium* Aiton, but other species are *V. myrtilloides* (Michx.), *V. boreale* I.V. Hall, and Alders, *V. pallidum* Aiton, and the hybrid *V. angustofloium* × *V. corymbosum* [2]. Maine accounts for 97% of the total US wild blueberry production [3].

Fruit set in wild blueberry is totally dependent upon animal pollinators. Bees are considered the most important pollinators of this crop [4,5,6]. There are more than 120 native bee species associated with wild blueberry in Maine [7]. The native bees that have been studied in wild blueberry are highly efficient and effective pollinators. They deposit multiple pollen grains on blueberry flower stigmas in each floral visit, have a short flower handling time [8,9], and the spatial pattern of foraging behavior maximizes pollen transfer between plants [10]. Commercial bumble bees, *Bombus impatiens* (Say), purchased by wild blueberry growers, are also effective pollinators [11,12].

Western honey bees [*Apis mellifera* (L.)] are not particularly efficient pollinators of wild blueberry on a per bee basis [5,9], but they are effective pollinators. Their effectiveness is due to the high densities of colonies deployed in wild blueberry fields [5], up to 20 hives per hectare. This high density of colonies deployed is despite a recommended hive stocking density of 5–10 hives per hectare. This is because growers perceive a great risk of bad weather during pollination, and therefore, they rent more hives than recommended as insurance. The recommended stocking density is variable because it depends upon the native bee community abundance [13]. In 2016, more than 80,000 hives were brought into Maine for wild blueberry pollination [Lund, unpublished data]. Although native bees provide about 20–30% of fruit set [5,14], wild blueberry growers rely heavily on honey bees to mitigate the risk associated with native bee community fluctuations in abundance from year to year [15]. Therefore, honey bees have been an increasingly important part of Maine wild blueberry production.

The dependence on honey bees by growers for pollination is not without risk, as stated above. Cold temperatures during bloom in Maine can decrease foraging activity [9,10]. The abundance of flowering plant species in and outside wild blueberry fields can decrease visitation to wild blueberry flowers [9,12,16]. In addition, unhealthy colonies can exhibit compromised foraging or collapse [17]. Honey bee colony health can be affected by parasites and pathogens, transportation stress, suboptimal nutrition, low genetic diversity, inadequate queen mating, and pesticide exposure [18,19,20,21,22,23]. Beekeepers throughout the world have been struggling to keep honey bee colonies alive since major declines and collapses were noted in the early part of this century with the occurrence of Colony Collapse Disorder (CCD) [24]. Colony loss since this time has been demonstrated to be a multiple stressor phenomenon involving the interaction of many factors [25].

### Objectives

Since honey bees are central to wild blueberry pollination in Maine, we sought to investigate their health during bloom. The specific objective of this study was to assess the potential health of migratory honey bee colonies brought into Maine wild blueberry fields to provide pollination services during bloom in 2014 and 2015. To determine colony health, colonies were sampled (see methods below) to estimate queen status (presence of queen and oviposition), preparation of queen supersedure, worker colony strength, worker colony strength rate of increase, sealed brood colony strength, sealed brood colony strength rate of increase, pathogen and parasite loads, and pesticide residues in trapped pollen, wax comb in the brood area, and stored bee bread.

## 2. Materials and Methods

### 2.1. Study Site and Colony Health Measures

This study was conducted in the major wild blueberry growing regions in Maine, USA. Our research was conducted in Hancock and Washington counties, in the towns of Alexander, Aurora, Cherryfield, Columbia, Deblois, Jonesboro, and the unorganized township T22, during the years 2014 and 2015. Beekeepers and blueberry growers were contacted by us for permission for us to sample hives that were brought into wild blueberry fields during the period of bloom (usually mid-May to mid-June). Several hive locations (clusters of hives) in sections of wild blueberry fields where the hives were set out during bloom were monitored each year. We monitored nine hive locations (clusters of hives within fields). We randomly selected three hives/location for monitoring in 2014 and nine locations (five hives/location) in 2015. In each field location, hives were on wooden pallets with 4–6 hives per pallet and the numbers of hives per field ranged from 60–200. Colony sampling was conducted three times during bloom. In 2014, the bloom was from 18 May to 13 June, and in 2015, the bloom was from 20 May to 16 June. Colony health was measured by sampling queen presence, egg laying, and the presence of supersedure cells, indicating preparation of queen supersedure. Sealed brood population was calculated by determining the percent area of wax comb on varying sized hive bodies with sealed brood and then converting to numbers of brood using published formulae [26]. Worker colony strength was calculated by determining the percent area of wax comb with worker bees on the surface on either medium or deep size hive bodies and then converted to numbers of workers using published formulae [26]. Health status was estimated by sampling each colony twice—1) shortly after hive deployment in blueberry fields and 2) just before bloom ended. A measure of population growth was calculated by determining the percent rate of change from the first sample to the second sample of both sealed brood and workers. Previous studies have found that colony strength and its rate of change is a reliable measure of colony health [27]. The formula we used to estimate the percentage rate of colony population change between the first and second samplings of the colonies is described below. Life stages sampled were works and sealed brood.

% rate of change = (colony strength at time t − colony strength at time t + 1) / (colony strength at time t) × 100

### 2.2. Pesticide Residues

In both 2014 and 2015, pesticide residue analysis was conducted on trapped pollen sampled during peak bloom on three colonies/location. Samples were pooled by location each year. Front entrance pollen traps (Anatomic Front Mount Pollen Trap^®^, Better Bee, Greenwich, NY, USA) were attached to each of the hive entrances without the trapping gate set for 24 h, allowing honey bee foragers to adapt to moving through the pollen trap. On the following day, the trapping gate on the pollen trap was closed, and pollen was trapped for 48 h, as foragers returned to the hive with pollen after foraging [28]. As honey bees with pollen moved through the pollen trap to enter the hive, a proportion of pollen was dislodged from their corbiculae and was collected in the pollen trap tray. Wax comb (ca. 100 cm^2^) was cut out and collected inside each hive adjacent to the brood area of three hives/location during late bloom. The wax comb samples were pooled by location. In addition (2015 only), 5–10 gm/hive of bee bread (stored pollen mixed with nectar) was extracted from wax comb cells with a spatula above the brood area. Bee bread was collected from each of the three hives/location and pooled by location. All residue samples (trapped pollen, wax comb, and bee bread) were transported at the end of the day of collection from the field to the laboratory in Orono, ME in insulated coolers (The Coleman Co., Inc., Golden, CO, USA) containing ice packs (Igloo Maxcold^®^ ice blocks, Igloo Co., Katy, TX, USA). Once at the laboratory, samples were stored at −80 °C in an ultra-freeze (Thermo Scientific^®^, Fisher Scientific, Hampton, NH, USA). Samples were shipped overnight to pesticide residue analysis laboratories when requested. In 2014, pesticide analyses of samples were conducted at the Connecticut Agricultural Experiment Station, New Haven, CT, USA, and in 2015, the analyses were conducted by the USDA National Science Laboratory, Gaston, NC, US. The Connecticut Agricultural Experiment Station analytical chemistry laboratory used high-pressure liquid chromatography analysis targeting 140 different pesticides, and metabolites after extracting the residue targets using a modified QuECHERs procedure [29]. More details of the procedures can be found in Ostiguy et al. [30]. The USDA National Science Laboratories in Gastonia, NC, USA, screened for 200 agricultural pesticides and metabolites using gas chromatography and high-pressure liquid chromatography with mass spectrometry. This laboratory also utilized a modified QuECHERs procedure for extracting residue targets from the various matrices. Limits of detection (LOD) mostly ranged from 5 to 25 ppb (maximum = 50 ppb) depending upon the matrix (pollen, wax comb, or bee bread) that residues were extracted from and the specific pesticide (e.g., pyrethrin LOD = 50 ppb). 

Limits of detection (LOD) ranged between 0.5 and 20 ppb depending on the matrix from which residues were extracted. Most of the compounds had a LOD of less than 5ppb, with 88 compounds at 1 ppb or less. However, the pesticides in the two laboratories’ screens differed and the detection levels differed for many of the same pesticides that were in the screens. Due to this, we chose to minimize laboratory bias by only selecting pesticides that both laboratories searched for and we only considered pesticide detections in trapped pollen and wax comb in 2014 and 2015 when we applied the higher level of detection (LOD) to both years of analytical results. For example, if the insecticide Phosmet in pollen had a LOD of 1 ppb by one laboratory, but 10 ppb by the other laboratory, we only considered detections for Phosmet in both 2014 and 2015 when concentrations were at 10 ppb or higher. We realize that this reduced the number of pesticide detections and the overall total concentration of residues by eliminating detection of low concentrations over the two-year period, but it provided a consistent benchmark for making comparisons of exposure and toxic risk between years. We also corrected the detections, as described above, for bee bread even though this matrix was only sampled in 2015.

A quantitative measure of pesticide risk was calculated from the residue data to determine if levels of exposure observed from pollen, wax, and bee bread affected colony health. Contact hazard quotients (HQ) were calculated using methods by previous authors [28,30], but because Oral LD50 estimates are (1) less available and (2) contact and oral HQs are highly correlated [28], we only estimated contact HQs for this study. To calculate the contact HQ, lethal dose 50th percentile values (LD50 in units of ppb) were compiled for all detected compounds based upon available literature and public databases (see [28,30] for information on databases). We used the LD50 values of parent pesticide compounds if LD50s were not available for metabolites [28,30]. Then, we divided the concentration of each pesticide quantified in trapped pollen, wax comb, or bee bread for a given location by the contact LD50 estimated for honeybees. Contact LD50 values reported in terms of μg/bee were converted to ppb relative to body weight (ng pesticide per g bee) by multiplying each value by a factor of 10,000; this is an approximate equivalent to 1000 ng per μg÷mean bee weight of 0.1 g [31,32]. An estimated HQ of 1.0 suggested that the exposure level by contact will result in 50% mortality to colony populations. An HQ greater than 1.0 represented an expectation of high proportions of mortality. Based upon these HQs, we assessed risk both at the individual pesticide compound level and pesticide use-group level, and also additively across all pesticides detected, which provides a measure of total colony risk. This total colony HQ assumes that effects due to pesticides are additive, and this is most likely not the case based upon studies showing synergy among pesticides in honey bees [33,34]. However, our use of an additive HQ was acceptable because total colony risk was used only as a relative measure of colony stress for comparing locations and years and not as an absolute estimate of acute mortality.

### 2.3. Parasites and Pathogens

Varroa mite, *Varroa destructor* (Anderson and Trueman), and tracheal mite, *Acarapis woodi* (Rennie) infestations were estimated by collecting ca. 400 workers (collected in 150 ml polyethylene cups), ca. 200 nurse bees from the brood area, and ca. 200 older field bees from honey and pollen frames in each of three (2014) or five (2015) hives at each location during peak bloom. Bees were preserved in ETOH (ethyl alcohol) until processing. In the lab, samples were counted, Varroa mite infestation was determined by counting the number of Varroa mites in solution and adjusting the number per 100 workers (using modified methods by Hendrickson) [35]. The same workers used for Varroa mite assessment were then dissected for tracheal mite assessment using the technique developed by Sammataro [36]. Varroa mite infestation was quantified as the number of mites per 100 worker honey bees and tracheal mite infestation was quantified as the percent of honey bee workers with tracheal mite detected by dissection. During the dissections for tracheal mite, we also looked for the parasitic phorid fly or “zombie fly,” *Apocephalus borealis* Brues, and quantified prevalence as percent worker bees with phorid parasites.

During the late bloom, samples of 200 workers from each hive at each location and year were taken from both brood comb and honey-filled comb to assess common honey bee viruses and other pathogens. Honey bees were transported at the end of the day of collection from the field to the laboratory at the University of Maine, Orono, ME, in insulated coolers containing ice packs. Once at the laboratory, samples were stored at −80 °C. In 2014, bees were shipped on dry ice to the National Honey Bee laboratory in Beltsville, MD, USA, for molecular detection of pathogens using qRT-PCR analysis. In 2015, samples were shipped on dry ice to the North Carolina State University Apiculture Queen Disease Clinic, NC, USA, for molecular detection of pathogens, again using qRT-PCR analysis. Each sample was screened for six known honey bee viruses in 2014: Israeli acute paralysis virus (IAPV), black queen cell virus (BQCV), Kashmir bee virus (KBV), chronic bee paralysis virus (CBPV), sacbrood virus (SBV), and deformed wing virus (DWV); and eight known honey bee viruses in 2015: acute bee paralysis virus (ABPV), chronic bee paralysis virus (CBPV), black queen cell virus (BQCV), deformed wing virus strains A (DWVA) and B (DWVB), Israeli acute paralysis virus (IAPV), sacbrood virus (SBV), and Lake Sinai virus (LSV). In 2014, one microsporidian pathogen—*Nosema ceranae* (Fries), and the Trypanosome group of pathogens at the order level—were screened. In 2015, *Nosema* (at the genus level), two Trypanosome pathogen groups, *Crithidia* (at the genus level), and other Trypanosomes not including *Crithidia* (at the order level) were screened. This Trypanosome was later determined to be only one species, *Lotmaria passim* Schwartz (Kinetoplastea). At both testing facilities, RNA was quantified on a Nanodrop Spectrophotometer, diluted, and then total cDNA was synthesized and used in a PCR reaction. The Normalization of final values to two reference genes (Ancr1 and AB Actin) values was performed in GeNorm [37] at the National Honey bee laboratory, and the genes (Apo28s, CamllK) were used for normalization at the NC laboratory. At both facilities, quantitative PCR was used to assess the presence of the pathogens and the intensity of infection (estimated number of viral copies). The normalized intensity of infection was logarithm (base 10) transformed for all analyses.

### 2.4. Statistical Analysis

Graphical visualization was used to illustrate observed colony health metrics and potential causal factors of colony health. Pearson linear correlation was used to determine positive or negative associations between pesticide residues (log(ppb)) in the three matrices in each year. Spearman correlation was used to assess if the different sampled matrices shared the same pesticide residues (presence/absence). Pearson correlation was also used to determine if associations existed between parasite and pathogen incidences. These analyses were only conducted for parasites and pathogens screened in both years. In these correlations for DWV as a variate, the DWV marker in 2014 was used, with the DWVA strain for 2015. Mixed models were used to test the effect of year on the colony percent rate of change, pesticide residues, parasites, and pathogens. The year was a fixed effect, and the residuals were assumed to have a random correlation structure within the year. A mixed model was also used to determine what potential causal factors determined colony health. The colony health metric, the sealed brood percent rate of change, was the dependent variable chosen for the analysis. Since the sealed brood percent rate of change was significantly related to the worker percent rate of change, we chose only to use one of the measures of colony health, the percent rate of change of sealed brood. The location-level HQ for pollen, wax comb, and both wax and pollen combined, and parasite measures (individual taxa, and all combined as total parasites) were fixed effects. The Year and its interactions with fixed effects were included in the models as random effects. Since pesticide analysis was conducted on samples pooled over colonies within a location, the causal factor analysis was performed with location-level data using the pooled and averaged values by locations for all dependent and independent variables. All correlations and mixed models were estimated with JMP statistical software [38].

## 3. Results

### 3.1. Colony Health

**Figure 1:**
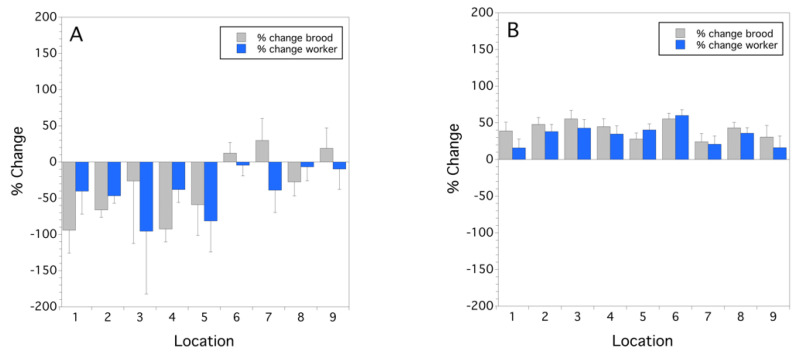
Mean colony population rate of change from initial colony strength over the bloom period per location for sealed brood and workers for 2014 (**A**) and 2015 (**B**); zero on the y-axis represents no change in sealed brood or worker colony strength during bloom. Error bars are standard errors of the mean (*n* = 3 and *n* = 5 for 2014 and 2015, respectively).

The change in colony strength was different in 2014, compared to 2015 (Figure 1A,B). Mean sealed brood rate of change was significantly less in 2014 (−33.8 ± 15.7 (s.e.) %) than 2015 (40.9 ± 3.8%) (*F*_(1,8)_ = 19.597, *p* = 0.002). This same pattern was also observed with worker populations, 2014 (−40.2 ± 10.7%) vs. 2015 (33.9 ± 4.8%) (*F*_(1,8)_ = 37.660, *p* < 0.001). Figure 1A shows that one-third of the locations had an increase in worker strength over the bloom period in 2014. In contrast, all of the locations in 2015 experienced an increase in colony strength during bloom (Figure 1B).

**Figure 2:**
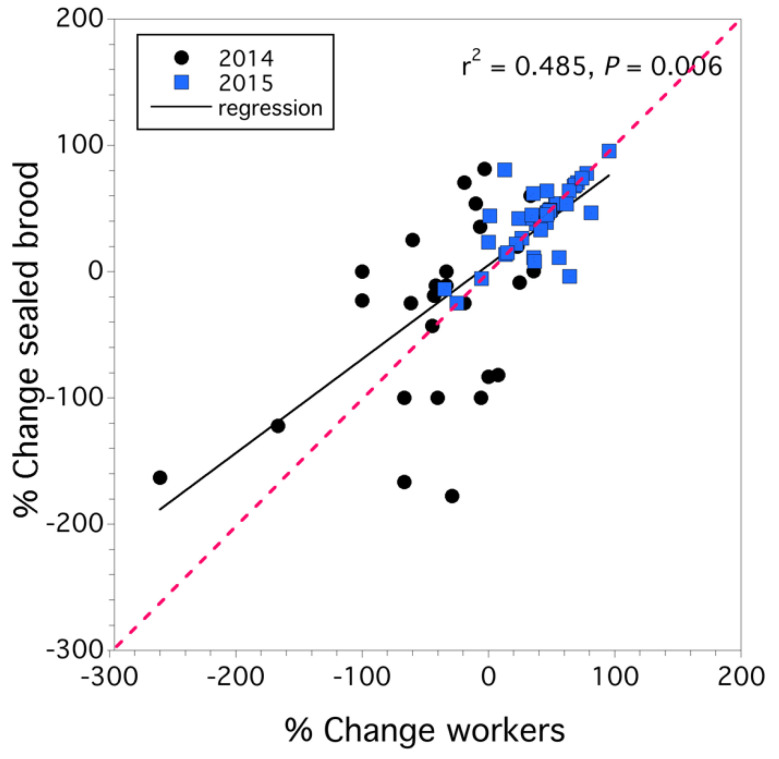
Linear relationship between percentage rate of worker colony strength and percent rate of change in sealed brood colony strength. Red dashed line is 1:1 slope.

Colonies that exhibited a lack of population increase did not appear to have significantly higher rates of queen supersedure (*p* > 0.05). Although, in 2014, 11.1% of the colonies sampled had undergone supersedure, and in 2015, supersedure occurred in 6.7% of the colonies sampled. The relationship between the percentage rate of change in sealed brood and that of workers for all 72 colonies sampled over the two years is shown in Figure 2. We found that the percent rate of change of workers determined the rate of change of sealed brood (*F*_(1,68)_ = 8.032, *p* = 0.006) and that while the effect of year was significant (*F*_(1,68)_ = 6.557, *p* = 0.013), there was no year x worker rate of change interaction (*F*_(1,68)_ = 0.304, *p* = 0.583). Overall, 48 percent of the variation in the percent rate of change in sealed brood was explained by the percentage rate of change in workers (Figure 2). As the rate of population decline increased (from 0% to −200%), workers declined faster than sealed brood, but when the rate of population change increased (>0%), workers increased at a higher rate than sealed brood (see regression line in Figure 2).

### 3.2. Pesticides

In 2014, 11 pesticides and their metabolites were detected in trapped pollen, and 21 compounds were detected in the wax comb. In 2015, 7 pesticides and their metabolites were detected in trapped pollen, 13 in the wax comb, and 9 in bee bread. The five pesticides or metabolites detected in the highest concentrations in both trapped pollen and wax comb in 2014, and trapped pollen, wax comb, and bee bread in 2015, are listed in Table 1. Fungicides, herbicides, insecticides, and miticides were represented in the residues with the highest concentrations. The concentrations listed in Table 1 have high variation between hives/location as reflected by the large standard errors relative to the mean concentrations.

**Figure 3:**
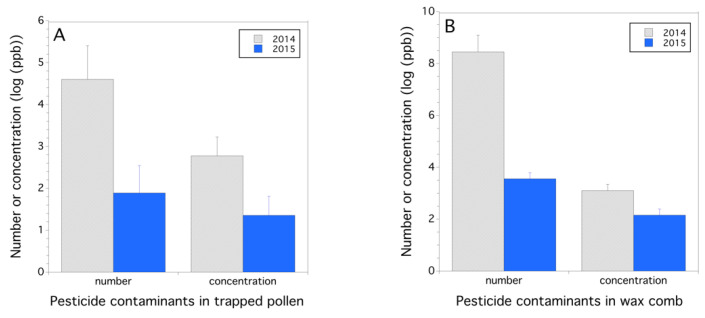
Concentration of pesticides and metabolites and number of pesticides detected per hive location in 2014 and 2015 in trapped pollen (**A**) and wax comb (**B**); concentration (ppb) is logarithm base 10 transformed.

Exposure to pesticides (parent chemical compounds and their metabolites) in trapped pollen was greater in 2014 than in 2015 (Figure 3A), both in numbers of pesticides/location (*F*_(1,15)_ = 7.124, *p* = 0.018) and total residue concentration (ppb)/location (*F*_(1,15)_ = 4.892, *p* = 0.043, mixed model with logarithm transformed ppb). Additionally, the number of pesticides detected in wax comb was greater in 2014 than in 2015 (*F*_(1,14)_ = 25.204, *p* < 0.001; Figure 3B). The log concentration (ppb) of pesticides in wax comb was higher in 2014 than in 2015 (*F*_(1,14)_ = 4.719, *p* = 0.048; Figure 3B). Trapped pollen is assumed to represent exposure from the flowers that honey bees are foraging on at the time of trapping, but because we found a high level of miticides in the pollen, this assumption has to be questioned and probably represents both pesticide contamination from inside and outside the hive. Pesticides contaminating wax is often thought to be an integration of pesticide exposure over a longer time period such as a growing season or several years until a new foundation replaces the older wax comb. Residues in bee bread in 2015 were similar to the residues in the 2015 trapped pollen. The pesticide numbers detected in bee bread were 3.0 ± 0.7, and the mean log concentration of residues was 2.1±0.4.

**Figure 4:**
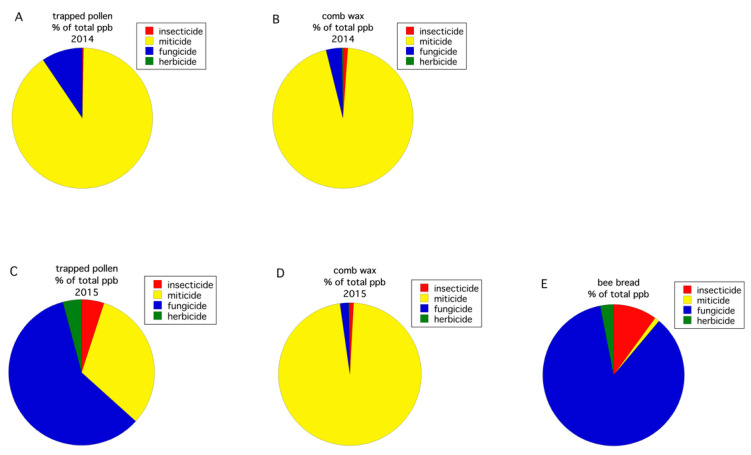
Percent composition of pesticide residue concentration (ppb) by use group in trapped pollen in 2014 (**A**), wax comb in 2014 (**B**), trapped pollen in 2015 (**C**), wax comb in 2015 (**D**), and bee bread in 2015 (**E**).

All routes of exposure (pollen, wax comb, bee bread) for the four pesticide use groups (fungicides, herbicides, primarily insecticides, and in-hive miticides), as reflected by residue concentration, were compared (Figure 4). In 2014, the pesticide group percent composition in trapped pollen (Figure 4A) was similar to what was detected in the wax comb (Figure 4B), with the majority of pesticides being miticides used to treat Varroa mite. Miticides comprised 90.2% of the total concentration of detected pesticide residues, in trapped pollen, and 95.1%, in the wax comb. However, in 2015, a different pattern was observed. In 2015, the pesticide group percent composition in trapped pollen was fungicides (56.6%) and, to a lesser extent, miticides (30.1%) in (Figure 4C), whereas pesticide detections in the wax comb (Figure 4D) was almost exclusively miticides (96.9%). The bee bread (Figure 4E) was similar to trapped pollen in 2015 (Figure 4C), with pesticide detections being primarily comprised of fungicides (85.8%). These data show that the makeup of contaminants, even at the level of pesticide use group, can vary greatly across years in the same cropping system and geographic region.

Pearson correlation analysis revealed that in 2014, the log (ppb) levels of pesticide residues in wax and pollen involving all compounds that were detected showed that residue concentrations found in trapped pollen were correlated with those in the wax comb (*r* = 0.329, *p* = 0.007, n = 66). When the presence or absence of detected compounds in wax comb and pollen were tested for correlation, we also found evidence for a significant correlation (Spearman’s ρ = 0.305 *p* = 0.013, n = 66). In 2015, no correlation was found between residues in trapped pollen and wax comb (*p* = 0.738). However, residues in bee bread were positively correlated with those in trapped pollen (*r* = 0.0.741, *p* < 0.007, n = 40) but negatively correlated with those in the wax comb (*r* = −0.577, *p* = 0.032, n = 42). The presence of specific residues of all the matrices in 2015 was correlated, including trapped pollen with the wax comb (ρ = 0.345 *p* = 0.009, n = 56), bee bread negatively correlated with trapped pollen (ρ = −0.577, *p* < 0.001, n = 40), and bee bread with the wax comb (ρ = 0.674, *p* < 0.001, n = 42). When both years were considered together, log residue concentrations and presence/absence of pesticide residues were correlated between trapped pollen and wax comb (r = 0.239, *p* = 0.008, n = 122; ρ = 0.222, *p* = 0.014, n = 122, concentration and presence/absence, respectively).

**Figure 5:**
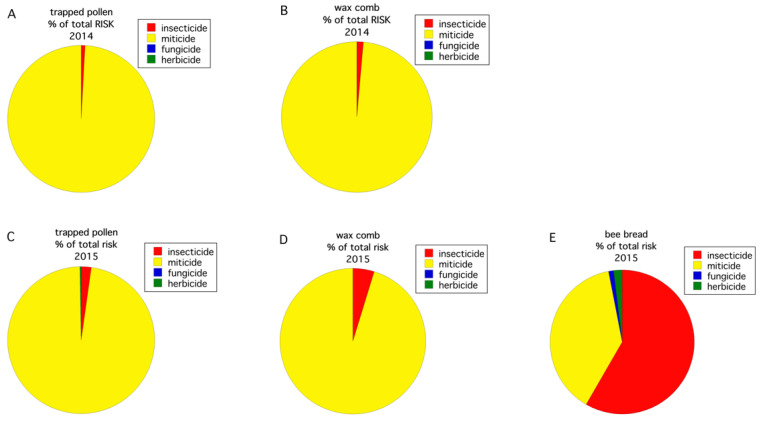
Percent composition of HQ by use group in trapped pollen in 2014 (**A**), wax comb in 2014 (**B**), trapped pollen in 2015 (**C**), wax comb in 2015 (**D**), and bee bread in 2015 (**E**).

The honey bee HQ (log transformed) varied significantly between the two years for trapped pollen (*F*_(1,15)_ = 6.029, *p* = 0.027). The HQ of the wax comb was only significant at α = 0.1 (*F*_(1,16)_ = 3.983, *p* = 0.063). Trapped pollen in 2014 had a higher HQ than that was found in 2015. The average HQ for trapped pollen in 2014 vs. 2015 was 2.38 ±2.01 vs. 0.06 ± 0.05. For wax comb, the HQ was also higher in 2014 than in 2015: 2.86 ± 1.94 vs. 0.38 ± 0.28. The trapped pollen HQ was significantly greater than the wax comb HQ in 2015 (F_(1,8)_ = 8.095, *p* = 0.022) but not in 2014 (F_(1,8.2)_ = 0.311, *p* = 0.592). Bee bread, only sampled in 2015, had an HQ of 0.02 ± 0.01 and was significantly different than the mean HQ of the wax comb (*p* = 0.011) but not trapped pollen (*p* = 0.926, Tukey HSD multiple comparisons). Figure 5 shows the percent composition by pesticide use group of the calculated HQ by year and route of exposure (i.e., trapped pollen, wax comb, bee bread). For both years, the risk to exposure of trapped pollen and wax comb is almost entirely due to miticides (Figure 5A–D), although insecticides contribute a measurable proportion of the total risk. Insecticides can contribute a disproportional amount of risk relative to concentration. As an example, in 2015, insecticides constituted 0.35% of total pesticide concentration (ppb) in the wax comb but only 4.8% of the total HQ (Figure 4D and Figure 5D), and 10.1% of total pesticide concentration in bee bread but 58.3% of the HQ (Figure 4E and Figure 5E). This is due to the high proportion of total ppb contamination by miticides (Figure 4A–D). Bee bread, in 2015, departed from the pattern of miticides, contributing to the majority of the HQ with the largest component of risk being insecticides (58.3%, Figure 5E), followed by miticides (38.7%, Figure 5E).

**Figure 6:**
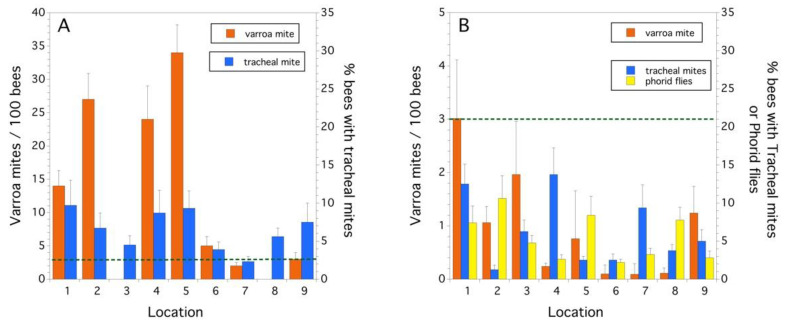
Infestation rates of Varroa mite, tracheal mite, and phorid flies in 2014 (**A**) and 2015 (**B**). Error bars are standard errors.

*Parasites and Pathogens.* Varroa mites were found in higher infestation levels in 2014, compared to 2015 (*F*_(1,8)_ = 6.509, *p* = 0.034), while tracheal mite infestation levels were not different between years (*p* > 0.05) (Figure 6A,B). Phorid fly eggs and larvae (*A. borealis*) were only found in 2015, but they were common at all locations (mean infestation/location = 5.5 ± 1.0%) (Figure 6B). The green dashed line in Figure 6A,B is the treatment threshold (in mites/100 bees) commonly recommended for Varroa Mite in the USA. [39]. In 2014, five of the nine locations had colonies with Varroa mite infestation levels greater than the threshold of 3 mites/100 honey bees [39]. In 2015, none of the locations exceeded this threshold.

**Figure 7:**
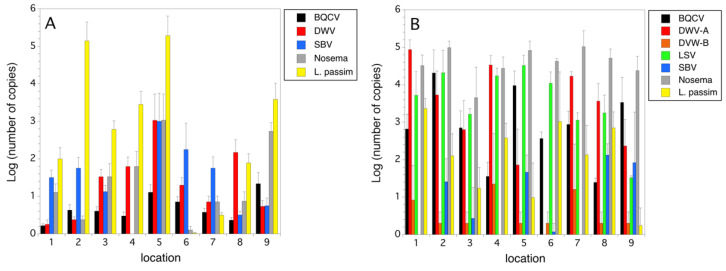
Pathogen incidence (logarithm base 10 (number of copies)) by hive location in 2014 (**A**) and 2015 (**B**). Error bars are standard errors.

In 2014, BQCV, DWV, and *N. ceranae* were detected at all nine locations, but virus incidences were at low to moderate levels relative to *L. passim* (Figure 7A)). Sacbrood virus and Trypanosome infections were detected at all but one location in 2014 (Figure 7A). Three viruses (KBV, IAPV, CBPV) were either at an extremely low prevalence and incidence or were absent in the honey bee populations we sampled. Due to this, we did not plot these three viruses (Figure 7A). In 2015, several viruses (ABPV, CPBV, and IAPV) were either not detected or were very low in prevalence and incidence (not plotted). Figure 7B shows that in 2015, five viruses and two unicellular pathogens were present at most of the hive locations. DWVA, LSV, and *Nosema* were the pathogens with the highest incidence. The *Nosema* genus level markers most likely represented either *N. ceranae* or *Nosema apis* (Zander, 1909). In both years *L. passim*, a low-level trypanosome pathogen, was quite common and had a fairly high incidence. When linear correlation analysis was performed between all log-transformed copy numbers for pathogen markers in both 2014 and 2015, we found three significant (Bonferroni corrected) associations in honey bees. Prevalence was high among all the commonly detected pathogens, and therefore, co-association within a colony was common. There were significant correlations between incidence of *Nosema* and BQCV (*r* = 0.682, *p* < 0.0001); *L. passim* and BQCV (*r* = −0.351, *p* = 0.001); and *L. passim* and DWV (*r* = 0.339, *p* = 0.001).

**Figure 8:**
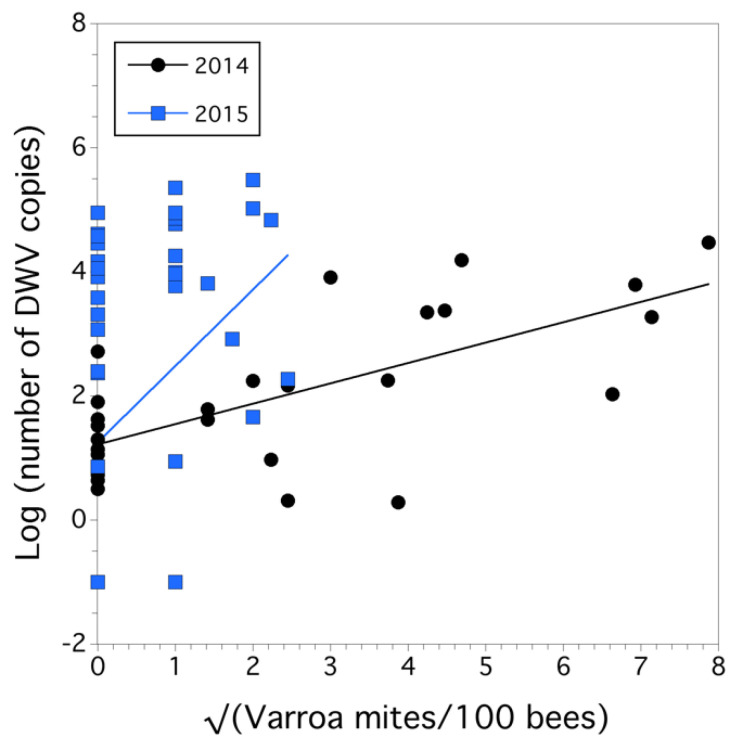
Relationship between Varroa mite infestation level (sqrt transformed) and the intensity of DWV infection (logarithm base10 (number of DWV copies) for each year.

We also found a causal relationship between the square root of Varroa mite infestation level (mites/100 bees) and the logarithm-transformed number of copies of the DWV marker (a proxy for disease intensity) (*F*_(1,73)_ = 13.883, *p* < 0.001), but there was also a significant year x sqrt (Varroa mite) interaction (*F*_(1,73)_ = 4.656, *p* = 0.034). The overall model was significant (*F*_(3,75)_ = 4.880, *p* = 0.004), *r*^2^ = 0.133 (Figure 8). The slope was significantly lower in 2014 (β = 0.328 ± 0.071) than in 2015 (β = 1.229 ± 0.46). 

*Causal effects of colony health.* Since percent change in sealed brood populations explained 48% of the variation (r^2^) in percent change in worker populations in a colony, we chose to model only percent change in sealed brood as a proxy for colony health. The percent change in sealed brood was best explained by a negative effect of the logarithm of Varroa mite infestation level (β = −78.128 ± 17.076, *F*_(1,14)_ = 20.933, *p* < 0.001) and a negative effect of the logarithm of trapped pollen HQ (β = −21.096 ± 9.118, *F*_(1,14)_ = 5.353, *p *= 0.036). Varroa mite infestation level and trapped pollen HQ were independent, not autocorrelated (*p *= 0.610). Scaled estimates of slopes suggested that Varroa mite was 1.7 times more influential in affecting percent change in sealed brood than the HQ of trapped pollen. Other factors, on an individual basis, also determined percent sealed brood, but they were either not significant (*p* > 0.05), or they were correlated with the causal factors that were the best predictors (e.g., log (the number of DWV copies) is determined by Varroa mite, as shown in Figure 8). The proportion of variance explained in the percent change in sealed brood by the model was high (conditional *r*^2^ = 0.578). The HQ for wax comb was not a predictor of colony health, and nor were any of the pathogens. When Varroa mite was not put in the model, tracheal mite infestation level demonstrated a trend toward being a significant predictor of negative colony health (*p *= 0.148), although Tracheal mite infestation level and Varroa mite infestation level were not correlated with each other (*p *= 0.159). *Nosema* was positively associated with percent change in the sealed brood (*r *= 0.651, *p *= 0.004), but we viewed an increase in *Nosema* as a result of an increase in colony strength and not a causal factor of percent change in the sealed brood because it did not support current knowledge about *Nosema* in that infection does not increase colony health, and hence, it was not included in a model to determine causality of colony health.

## 4. Discussion

Wild blueberry is an obligate insect-pollinated plant, mostly dependent upon bees [5,40]. Migratory honey bee hives are heavily used to supplement or replace the pollination service by native bee species [5,41]. There has been concern by both honey beekeepers and wild blueberry growers about the health of colonies that are brought in for pollination services each year. In our study, we found that colony health varies over years. 

In 2014, colony strength declined over the bloom period. In 2015, colony strength increased during bloom. Although wild blueberry plants resupply nectary tissues daily during bloom, these plants can also exhibit nectar dearth during bloom [42]. Many migratory colonies brought to wild blueberry for pollination are fed sugar syrup during bloom, and therefore, we suspect that the differences we observed between years were not due to starvation. However, the feeding of the colonies that we studied was not being conducted during our sampling; hence, we do not know which colonies were being fed and which ones did not receive sugar syrup during bloom. Pollen nutritional content of wild blueberry has been shown to be suboptimal for honey bees [43], but honey bees usually do not collect a high percentage of blueberry pollen while foraging, and most of their dietary intake of pollen comes from other plant species surrounding wild blueberry fields [12]. We speculate that the nutritional quality of pollen would not fluctuate annually in a way that would explain the observed differences in colony health between 2014 and 2015, but we have no evidence of this.

We found that colony health over the two-year study period was best described by Varroa mite densities and the HQ estimated from trapped pollen pesticide residues (both logarithmically transformed). The residue concentrations in 2014 were higher than in 2015, but in both years, the amount of miticide and miticide metabolites found in both pollen and wax comb was high. In this study, we have assumed that Amitraz, Coumaphos, Fluvalinate, and their metabolites were all due to the use of these compounds as miticides to control Varroa mite. This is based upon the supposition that formulations of these compounds are currently registered for Varroa mite control, and because none of these pesticides were or are recommended for use in wild blueberry insect pest management [44], and as far as we know, they have never been used by wild blueberry growers for crop pest management. The dominance of miticides in pollen and wax comb has been reported in a large-scale apiary study conducted in Spain [45]. These miticides are toxic to honey bees at high doses, which were detected in this study [30]. In 2014, miticide levels in hives were exceptionally high, and yet Varroa mite levels were also extremely high. This situation might reflect Varroa mite’s resistance to Amitraz and Coumaphos miticides. In the US, resistance was first observed for both miticides in the late 1990s and early 2000s [46,47], although it can be seen that a decade and a half after the first reports of resistance, these miticides were still being heavily used.

Even though we found Varroa mite and the trapped pollen residue HQ to be significant predictors of percent colony strength change during bloom, one must still be cautious in concluding that the cause of the differences in colony health was due to only these two factors. Pesticide residues in the hive result in a complex dynamic and one measure, trapped pollen HQ, may not adequately capture the mechanisms at play and subsequent health risk to the colony. We are aware that our measure of risk to honey bee colonies is crude. We only estimate contact risk based upon the LD_50_ response to pesticides in workers. Our approach did not capture toxicities of “inert“ ingredients used in pesticide formulations that have been shown to have detrimental behavioral and physiological effects on honey bees [48]. It also did not capture the oral risk, which cannot be predicted from contact risk [30], or larval sensitivity to pesticide exposure [49], or synergy among mixtures of pesticides, which is the norm in the hive environment [50].

It is difficult to make predictions about the environment outside the hive with trapped pollen. Our initial assumption, along with other authors of several published studies [51], is that trapped pollen represents the current contamination of pollen and floral surfaces in the foraging territory of the honey bee colony (in this study, blueberry fields). However, this might not be a valid assumption for all residues detected. We are suspicious that the high level of miticides that would be used for Varroa mite control detected in pollen would be an independent measure of floral contamination. However, it could be the case that honey bee body surfaces, contaminated with miticide after a recent miticide treatment, contaminated pollen by direct body contact of the contaminated honey bees with floral surfaces or that contamination of pollen occurred when previously contaminated honey bees groomed the pollen off of their bodies and packed the pollen in their corbiculae [52]. In a similar manner, bee body contact with floral surfaces is the suggested mechanism of the transfer of honey bee parasites and pathogens to native bees [53].

Wax comb residues were initially assumed to be predictive of colony health since they represent the integral of incoming contamination over time (minus degradation). However, in our study, trapped pollen HQ was a better predictor of colony health. This may be explained by differences in actual exposure (food vs. contact through comb). This is difficult to measure but has been demonstrated with differences in outcomes to queens exposed to different sources of pesticides during development [54]. In addition, HQs of trapped pollen and wax comb were not correlated (*p* = 0.429). The HQs of bee bread, the processed food of larvae, was also expected to be a good predictor of colony health, especially sealed brood percent change, but we only had data from 2015, and therefore, this metric could not be adequately tested. However, the HQs of bee bread were significantly correlated with the HQs of trapped pollen in 2015 (*r* = 0.879, *p* = 0.002); hence, the risk due to contaminated bee bread may also be a good predictor of colony health in future studies.

Pesticide residues can have acute effects on individual honey bees and also colonies [19]. However, exposure can also result in more chronic conditions [55]. Symptoms of pesticide exposure can be the death of individuals in the colony [19], reduction in colony growth rate [56], reduction in queen productivity, increase in supersedure or queen loss [54], reduction in cognition and sensory modalities [57], and repellency of floral resources to foragers [58].

While colony losses have been shown to be caused by exposure to pesticides, especially insecticides, fewer studies have shown that miticides used to treat Varroa mite can have negative effects on colony strength. Johnson et al. [59] showed that interactions between miticides can result in highly toxic responses in honey bee workers. The use of HQs has been used to assess exposure and potential colony effects [30,31], but only a few studies have used these metrics with success to explain colony losses or declines in colony strength over time [60,61].

Honey bees in the US are often characterized by a high diversity and heavy load of pathogens and parasites [62]. We found this to be the case in both years of our study. Both tracheal mites and Varroa mites were abundant but not equally across all locations. Molecular markers for five viruses were common with relatively high copy numbers, while markers for four viruses were either not detected or not prevalent and usually were represented by low copy numbers. The recently (2011) discovered Lake Sinai virus [63] was at high prevalence (present in all sampled colonies in all locations) in 2015 (but not assayed for in 2014). Other studies have found similar high prevalence and incidence along with evidence suggesting that virulence can be high [64,65].

We were surprised to observe infestation of the parasitic phorid, *A. borealis* in 2015 at all sites, ranging from 2.2 to 10.6% parasitism (average 5.5%). We did not detect it in 2014. In 2012, this parasite was detected in commercial colonies in South Dakota; San Francisco, California; and the Central Valley of California with parasitism levels at 12–38% [66]. It has been reported in the published literature in the US since our study in 2015 but at low parasitism levels of 1–5% [67]. Whether this parasite is still common in commercial honey bee colonies in the US is unknown. It has also been reported as a new parasite of the honey bee in Belgium [68] and Egypt [69]. Another new pathogen of fairly high prevalence and incidence (copy number) in our study was the trypanosome, *L. passim*. This pathogen was described in the western honey bee in the US in 2015 [70], and it has since been found to be detrimental to colony health [71]. With this rich diversity of pathogens and parasites, why was Varroa mite found to be the only significant causal factor in colony health? We speculate that because of the high level of co-occurrence of many of these pathogens in a colony and with the presence of the immune system compromising Varroa mite [72], pathogens become highly prevalent and abundant. Therefore, it is difficult to tease out a single causal pathogen agent. Additionally, at a hierarchical level, high Varroa mite infestation in a colony represents a colony that has severe multiple pathologies of potentially different composition, which ultimately can lead to a decline in colony health. The only constant or “Holy Grail” appears to be Varroa mite.

## 5. Conclusions

The health of migratory honey bee hives brought to wild blueberry for pollination was observed to vary over the two years that we conducted the study. Varroa mite infestation levels and pesticide residues in pollen (as measured by an HQ) accounted for 57.8% of the variance in colony percent population growth rate during bloom. In general, pesticide residues other than miticides for the control of Varroa mites were common but were not responsible for explaining a significant proportion of the variation in the percent rate of change of sealed brood (*p* < 0.498) when miticides were taken out of the HQ. Tracheal mite and many of the pathogens were common in both years but were also not significant causal factors of colony health. Therefore, it appears that Varroa mite is the main factor responsible for the colony health of migratory hives brought in to pollinate wild blueberry. This is because the trapped pollen HQ we identified as a causal factor is most likely the result of Varroa mite control prior to and during pollination. Varroa mite appears to have both direct and indirect effects on western honey bee colony health in wild blueberry during pollination.

## Figures and Tables

**Table 1:** Pesticide residues in highest average concentrations (ppb/location) in trapped pollen, wax comb, and bee bread, in 2014 and 2015.

Year	Trapped Pollen (ppb) ^1^	Wax Comb (ppb)	Bee Bread (ppb)
2014	DMPF ^2^ (3853.7 ± 3262.9) M ^3^ tau-Fluvalinate (462.3 ± 280.8) M Chlorothalonil metabolite ^4^ (257.9 ± 118.9) F Boscalid (47.9 ± 81.7) F Propiconazole (24.8 ± 9.9) F	tau-Fluvalinate (3849.0 ± 3756.6) M DMPF ^3^ (1458.6 ± 1219.4) M Chlorothalonil metabolite ^4^ (107.4 ± 73.5) F Coumaphos (54.1 ± 29.8) M Propiconazole (54.0 ± 46.9) F	Not sampled in 2014
2015	Fludioxonil (98.8 ± 68.5) F DMPF ^3^ (95.5 ± 74.0) M Cyprodinil (85.7 ± 60.1) F Phosmet (16.3 ± 12.6) I Sethoxydim (13.3 ± 7.4) H	DMPF ^3^ (588.9 ± 471.5) M Tebuconazole (6.0 ± 6.0) F Coumaphos (5.9 ± 1.5) M tau-Fluvalinate (5.3 ± 5.3) M Pyrimethanil (2.2 ± 2.2) F	Fludioxonil (621.1 ± 614.9) F Cyprodinil (475.8 ± 468.1) F Phosmet (143.0 ± 114.7) I Chlorothalonil metabolite ^4^ (117.8 ± 116.5) F Sethoxidym (42.3 ± 39.6) H

^1^ Mean ± s.e. concentration (ppb) averaged over location. ^2^ DMPF (2,4 dimethylphenyl formamide) is a metabolite of the miticide Amitraz for Varroa control. ^3^ Use Category: F = fungicide, H = herbicide, I=insecticide, M = miticide for Varroa mite control. ^4^ 4-hydroxychlorothalonil is a metabolite of Chlorothalonil fungicide.

## Data Availability

Data can be obtained upon request from the author Francis Drummond.
